# A Standalone Capacitive Tactile Fingertip Module for Multi-Point Contact Sensing in Robotic Grasping

**DOI:** 10.3390/s26154756

**Published:** 2026-07-27

**Authors:** Suncheol Kwon, Dongwoo Nam, Wonseok Shin, Bummo Ahn

**Affiliations:** 1 Korea Institute of Industrial Technology, Sangnok-gu, Ansan-si 15588, Republic of Korea; skwon@kitech.re.kr (S.K.);; 2 Human in Motion Robotics Asia Inc., Gangnam-gu, Seoul 06224, Republic of Korea

**Keywords:** flexible capacitive sensor, tactile sensing, pressure/force sensing, robotic hand, standalone module, sensor integration

## Abstract

**Highlights:**

**What are the main findings?**

- A self-contained capacitive tactile fingertip module was developed by integrating a 3 × 1 flexible sensor array, capacitance-to-digital conversion, Bluetooth communication, and an onboard battery within a compact and replaceable fingertip housing.
- Individual capacitive sensors showed a near-linear response to normal compression up to 2.5 N and the assembled module produced distinguishable multi-point capacitance changes during robotic grasping of three cylindrical objects.

**What are the implications of the main findings?**

- The module can enhance robotic grippers with wireless tactile sensing without external cabling or major modifications to the hand controller.
- The distributed capacitance signals provide compact contact-location and relative local-load information for future tactile-feedback grasp monitoring and control.

**Abstract:**

Tactile sensing can enhance robotic grasping by providing contact information unavailable from vision or control signals alone. However, implementing tactile sensing in robotic hands is often constrained by external wiring, data-acquisition hardware, power requirements, and limited fingertip space. This study presents a self-contained capacitive tactile fingertip module for adding wireless multi-point contact sensing to robotic grippers. The module integrates a 3 × 1 array of thin flexible capacitive sensors, a capacitance-to-digital converter, a Bluetooth-enabled microcontroller, and an onboard battery within a compact fingertip-shaped housing. It can be mounted in place of an existing fingertip and operates independently of the robotic hand controller. Individual sensors characterized before module integration responded near-linearly to normal compression up to 2.5 N, with an approximately 8% relative capacitance change at 2.5 N and R^2^ = 0.99, and were evaluated over 100 repeated compression cycles. In proof-of-concept grasping tests with an empty PET bottle, a water-filled PET bottle, and a water-filled aluminum tumbler, the assembled module produced distinguishable capacitance changes at the mid- and proximal-position sensors, providing relative information on contact location and local loading rather than calibrated force. These results demonstrate the feasibility of wireless tactile sensing in robotic grasping using a compact standalone fingertip module.

## 1. Introduction

Tactile sensing is a fundamental modality for physically interactive robots [1]. Tactile information can be collected from a rich set of signals generated when robots come in contact with their environment and be used to enhance their physical intelligence. Particularly, tactile information plays an important role in performing challenging manipulation tasks that cannot be completed based on visual information alone [2]. Occlusion from robotic grippers can also limit in-hand object-pose estimation. Dikhale et al. [3] showed that pose estimation of an in-hand object can be more efficiently and accurately achieved by combining vision and tactile sensor data. Tactile data also provide crucial information when moving or manipulating objects of unknown weight, such as a cup filled with water [4].

Existing tactile sensing systems for robotic hands have been developed using various transduction and integration strategies. Fiber Bragg grating (FBG) sensors have high sensitivity and have been applied to robot finger tactile and sliding sensing, but optical interrogation hardware can limit complete fingertip-level integration [5,6,7]. Soft tactile sensors based on piezoresistive and capacitive transduction mechanisms have also been widely investigated because of their simple structures and compatibility with flexible materials [8,9,10]. Camera-based or vision-based tactile fingertips can provide rich spatial information and contact reconstruction, but they generally require optical components, illumination, image acquisition, and image processing [11,12]. Distributed electronic skins and tactile arrays can increase spatial coverage and provide multi-point contact information; however, increasing the number of sensing elements also increases channel count, wiring complexity, readout electronics, and integration burden [13,14,15,16].

Recent studies have further demonstrated tactile arrays on robot grippers, photoelastic tactile fingertips, FBG-based sliding sensors, curved flexible tactile sensors, and EIT-based soft fingertips, highlighting the continuing trade-off between tactile resolution and system-level integration [17,18,19,20,21,22,23,24]. Therefore, tactile fingertip modules should be compared not only by sensing principle or spatial resolution, but also by sensor density, module mass, onboard readout, onboard power, wireless communication, wiring dependence, and mechanical replaceability.

As summarized in Table 1, high-density tactile fingertips and vision-based tactile sensors provide richer spatial information, whereas their integration often requires cameras, illumination, external readout hardware, multiple sensing channels, or wired data/power connections. Standalone sensing modules are useful when sensing, readout, communication, and power must be integrated close to the contact interface [25,26,27,28]. For robotic fingertips, this requirement is particularly important because fingertip space is limited and external wiring can interfere with grasping and hand integration.

The present study intentionally prioritizes a lower-resolution but self-contained architecture. The proposed module integrates a 3 × 1 flexible capacitive sensor array, capacitance-to-digital conversion, microcontroller-based processing, Bluetooth communication, and onboard power within a compact fingertip-shaped housing. In this study, fingertip replaceability refers to the ability of the module to be mounted in place of the original fingertip of the tested robotic hand without electrical connection to the hand controller. Compatibility with other robotic hands would require compatible fingertip geometry or an appropriate mechanical adapter. Therefore, the main contribution of this work is system-level integration and electrical independence for wireless multi-point contact sensing, rather than high-resolution tactile imaging or calibrated force measurement by the assembled fingertip.

Based on this positioning, the contributions of this work are as follows:

(1) The design and fabrication of a compact standalone fingertip module integrating a 3 × 1 flexible capacitive tactile sensor array, capacitance conversion, microcontroller-based processing, Bluetooth communication, and onboard power;
(2) Bench characterization of the flexible capacitive sensors under static and low-frequency repeated compression up to 2.5 N;
(3) A proof-of-concept robotic grasping demonstration showing location-dependent relative capacitance responses while operating independently of the robotic hand controller.

## 2. Capacitive Sensor Fabrication and Pre-Integration Characterization

**Figure 1:**
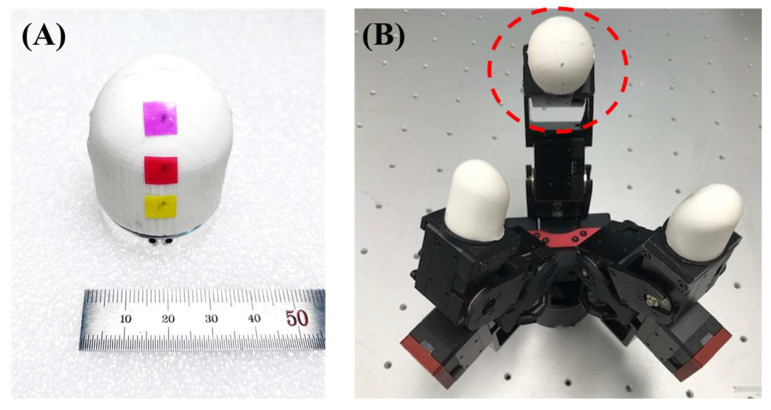
(**A**) Developed standalone tactile sensing module: the tri-color markers indicate the approximate positions of the three capacitive sensors embedded beneath the silicone fingertip skin. (**B**) Standalone tactile sensing module mounted on a three-fingered robotic hand: the module was installed on the tip of one finger (in red dashed circle).

**Figure 2:**
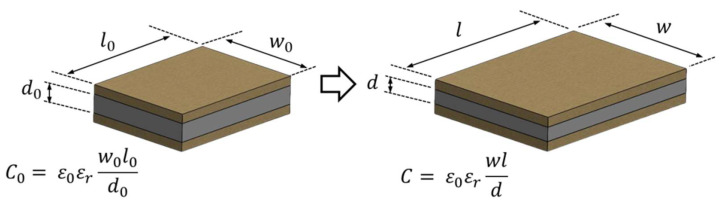
Basic principle of a capacitive sensor: *C* denotes capacitance; *w* and *l* denote the width and length of the conductive parallel plates, respectively; *d* denotes the thickness of the dielectric layer; ε_0_ and ε_r_ denote the vacuum permittivity and relative permittivity of silicone rubber, respectively; and subscript 0 indicates the initial state.

Robotic fingers can grasp a variety of shapes. Sensors, mounted on a robotic fingertip, must be durable and simple, and practical in structure. Herein, we embedded our robotic fingertip with three capacitive sensors, as shown in Figure 1. Practical sensors can be fabricated with a three-layer capacitive structure composed of a single dielectric layer between two conductive layers. Because the capacitance of the sensor changes as the area of the dielectric layer or the distance between the two conductive layers changes, change in the capacitance, as the volume of the dielectric layer changes, can be measured with mechanical compression by an external force; this is shown in Figure 2. In other words, tactile sensing due to physical contact can be inferred by measuring the change in the capacitance.

**Figure 3:**
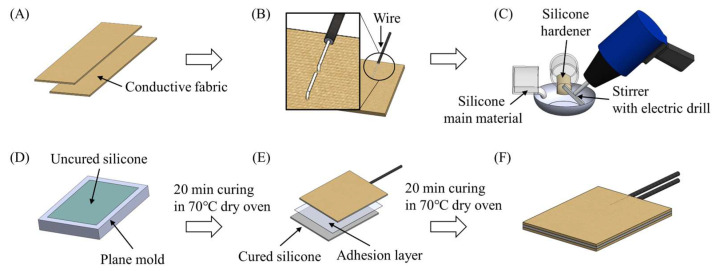
Fabrication process of the capacitive sensors using conductive fabrics and silicone rubber.

**Figure 4:**
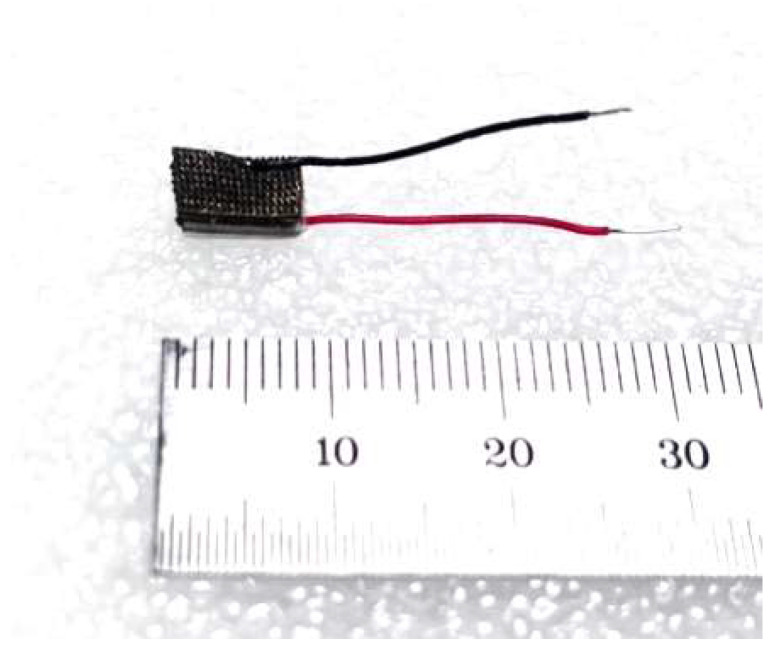
Fabricated capacitive sensor.

We fabricated capacitive sensors using conductive fabrics (WY-CF005, Wooyang Advanced Material Co., Daegu, Republic of Korea) and silicone rubber (Ecoflex™ 00-30, Smooth-On Inc., Macungie, PA, USA). A 0.5-mm thick conductive fabric was cut to size (Figure 3A) and connected to wire leads (Figure 3B). To make the dielectric layer, we mixed two polymer components of Ecoflex 00-30 silicone in a 1:1 volume ratio and stirred the mixture for 1 min using an electric drill (Figure 3C). The silicone mixture was poured into a dielectric layer mold of 1.0-mm thickness. The mold was degassed under 0.7-bar vacuum inside a vacuum chamber (MadeLab, Hanam, Korea) for 2 min before being cured in a dry oven (SH Science, Bucheon, Republic of Korea) at 70 °C for 20 min (Figure 3D). The cut fabric was attached to either side of the cured dielectric layer, and the resulting 3-layer device was cured in a dry oven at 70 °C for 20 min (Figure 3E). Final dimensions of the fabricated individual sensor (Figure 3F and Figure 4) were 8, 6, and 2.5 mm in width, length, and thickness, respectively. Before mechanical characterization, the initial capacitance, C_0_, of each sensor was measured under no external load; the representative C_0_ was 10.550 ± 0.487 pF.

In a robotic fingertip, normal compression was considered as the primary loading condition, while grasping the object with minimal change to the longitudinal force. Therefore, the thickness change of the dielectric layer, and not the longitudinal length change, is the most important factor affecting the change in the sensor capacitance upon mechanical stimuli. We conducted experiments to identify the correlations among the contact force exerted on the sensor when compressing an object, the capacitance, and the compression displacement. The characterization tests applied normal compression only; lateral or shear loading was not applied in the sensor-level tests.

The compression tests in this section were conducted on individual capacitive sensors before their integration into the fingertip module. The fully assembled fingertip, including the silicone skin, was not calibrated under controlled external loading. Therefore, the calibration relationship obtained from the individual sensors was not used to convert the capacitance responses of the assembled fingertip into absolute force values.

Static compression tests were performed by applying calibrated weights corresponding to 0.5, 1.0, 1.5, 2.0, and 2.5 N to the active sensing area. At each force level, the load was maintained for 10 s, and capacitance was recorded at 100 Hz. A total of 1000 capacitance samples were collected for each force level, and the mean and standard deviation were calculated. The initial capacitance, C_0_, was defined as the mean capacitance measured before applying an external load. Linear regression was performed using the mean relative capacitance change, ΔC/C_0_, at each force level. The results of the linear regression showed a very high linearity (fitted sensitivity of 3.36% ΔC/C_0_ per N, coefficient of determination, *R*^2^ = 0.99). At 2.5 N of applied force, the relative change in the capacitance was 8% from the initial state with no external weight applied.

(1) $$C_{r}=\frac{∆C}{C_{0}}\times 100=\frac{{C-C}_{0}}{C_{0}}\times 100\;(\%)$$

where *C_r_*, *C*_0_, and *C* denote the relative capacitance, initial capacitance, and capacitance due to compression, respectively.

**Figure 5:**
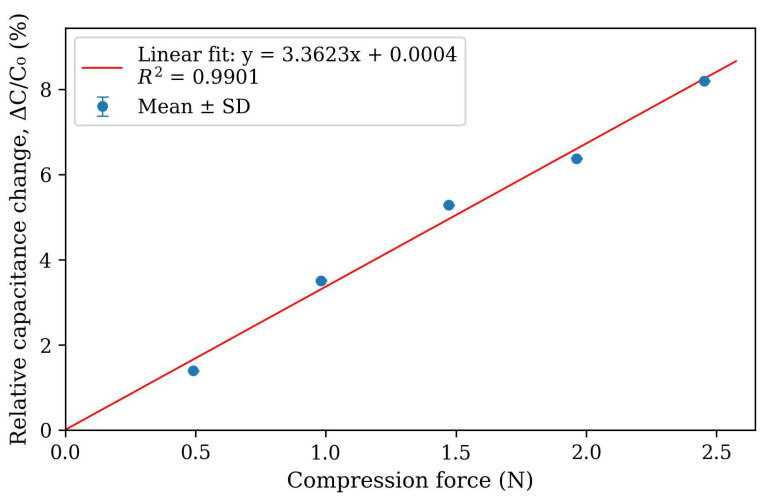
Linear correlation between compression force on the capacitive sensor and the relative change, ΔC/C0 (%), in the sensor capacitance. Markers indicate the mean values at each force level, error bars represent the standard deviation of the recorded samples, and the red line represents the linear regression fitted to the mean values. Error bars are smaller than the marker size because the standard deviation of the 1000 samples at each force level was small.

The linear regression in Figure 5 was used as a local empirical approximation over the tested normal-force range of 0.5–2.5 N, rather than as a global response of the capacitive sensor. To quantify the deviation from linearity, the maximum absolute deviation from the fitted line was 0.33% ΔC/C_0_, and the RMSE was 0.234% ΔC/C_0_. The standard deviation at each force level was 0.01% ΔC/C_0_, which is why the error bars are partially hidden by the markers in Figure 5. Error bars in Figure 5 represent the standard deviation of the 1000 samples.

**Figure 6:**
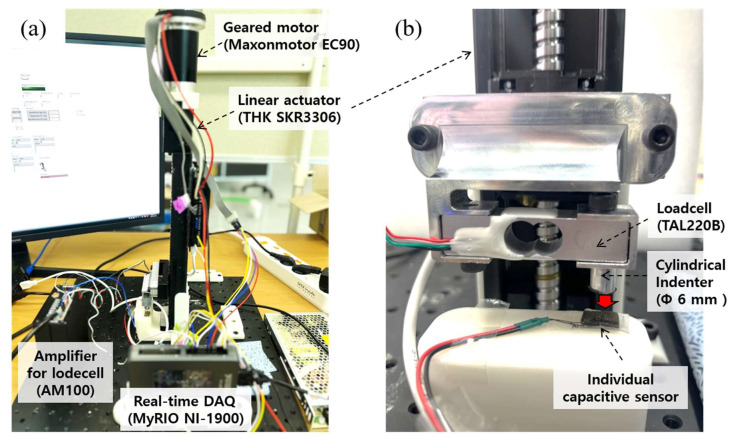
Experimental setup for normal compression testing of the individual capacitive sensor. (**a**) Custom linear actuator system consisting of a brushless DC motor, linear stage, and single-axis load cell. (**b**) Enlarged view of the cylindrical indenter aligned with the active sensing area of the capacitive sensor. The red arrow indicates the normal compression direction.

**Figure 7:**
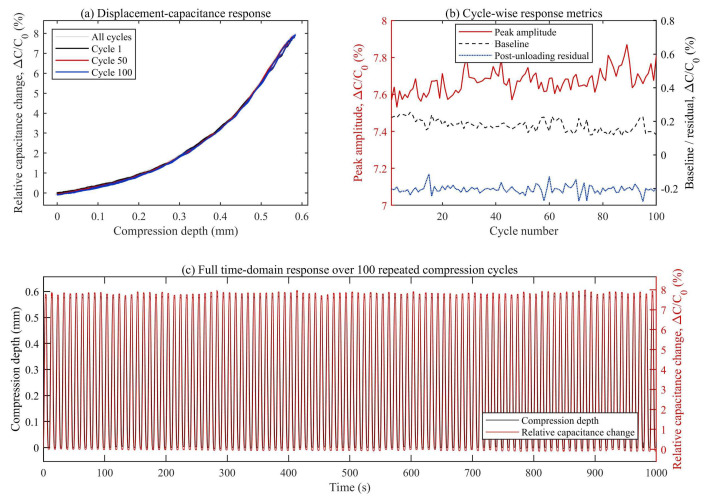
Low-frequency repeated-compression response of the capacitive sensor over 100 cycles. (**a**) Superimposed displacement–capacitance responses over 100 cycles, with Cycles 1, 50, and 100 highlighted. (**b**) Cycle-wise peak amplitude, baseline, and post-unloading residual response. (**c**) Full time-domain response of compression depth and relative capacitance change over 100 repeated compression cycles.

For low-frequency repeated-compression tests, we measured the relative change in the capacitance over 100 repetitions of compression, while measuring the contact force on a customized linear actuator with a loadcell attached, as shown in Figure 6. A cylindrical indenter with a diameter of 6 mm was aligned with the active sensing area and used to apply normal compression. The actuator generated trapezoidal smooth waveform displacement between the contact-onset position and a maximum compression depth of approximately 0.58 mm for 100 cycles at 0.1 Hz. For this experiment, a custom-built testbed included a linear actuator (SKR3306C, Samik, Suwon, Republic of Korea), a loadcell capable of measuring compression force in a single axis, and a cylindrical indenter to compress the capacitive sensor. The change in the compression depth by position control of the brushless DC motor (EC90 with reduction gear ratio of 113:1, Maxon motor, Sachseln, Switzerland), output of the loadcell (TAL220B, Sparkfun, Niwot, CO, USA), and change in the capacitance of the sensor were recorded by a real-time controller (NI-1900 myRIO, National Instruments, Austin, TX, USA) via LabVIEW 2018. To allow for comparative analysis with static test results, linear displacement compression was applied until the maximum applied load in the static test and compression force measured by the loadcell in the repeated-compression test were of similar magnitude. Using the linear actuator, we repeated the compression from an initial depth where contact was initiated to approximately 0.58-mm depth, and a maximum contact force of about 2.5 N was measured, corresponding to approximately equivalent to a 250 g static load. Similar to the static test, the maximum relative capacitance varied by 8%. While the compression force–capacitance plot shown in Figure 5 indicates capacitance linearity against the compression force, the compression displacement–capacitance plot shown in Figure 7 presents a curve with minimal hysteresis against the compressed depth. The fact that dielectric thickness is the denominator in the fundamental capacitance equation, explains why the change in the dielectric thickness due to linear compression is represented by the curve in Figure 7. These results indicate that the sensor maintained a stable short-term response over 100 repeated compression cycles. This test was intended to evaluate short-term cyclic repeatability under a slow compression condition and was not designed to characterize the frequency response or dynamic bandwidth of the sensor.

For the repeated compression test, the cycle-to-cycle response was analyzed from the 100-cycle capacitance trace. Peaks were detected from the capacitance signal, and individual cycle boundaries were defined as the midpoints between adjacent peaks. For each cycle, the peak response was extracted as the maximum capacitance value. The pre-loading baseline was calculated as the mean of the lowest 10% of samples before the peak response, and the post-unloading baseline was calculated as the mean of the lowest 10% of samples after the peak response. The response amplitude was defined as the difference between the peak response and the pre-loading baseline. Cycle-to-cycle variability was quantified using the coefficient of variation of the response amplitude. Baseline drift was calculated from the linear fit of the pre-loading baseline over 100 cycles and normalized by the mean response amplitude. Post-unloading residual response was defined as the difference between the post-unloading and pre-loading baselines, normalized by the response amplitude of each cycle. Across 100 repeated compression cycles, when normalized by the pre-loading baseline of each cycle, the response amplitude was 7.70 ± 0.07%, with a coefficient of variation of 0.86%. The baseline drift estimated from a linear fit over 100 cycles was −0.078%, corresponding to −0.99% of the mean response amplitude. The post-unloading residual response was -0.02 ± 0.02%.

## 3. Standalone Tactile Sensing Module

Our goal was to develop a standalone robotic fingertip module that provides tactile information. Consequently, the fingertip module had to satisfy the following requirements: (1) it must be capable of providing information on what is in contact with the external environment at two or more locations; (2) it must be capable of operating independently of the existing robotic system; (3) it must be capable of transmitting measured information to the robotic system or the external environment; and (4) it must be of a size and material compatible with existing fingertip modules.

To this end, we designed a standalone module that (1) has three capacitive sensors, (2) has a built-in battery for independent operation, (3) transmits the measured data via Bluetooth wireless communication, and (4) has a dome-shaped structure, 45 g in weight, 34 mm in diameter, and 42 mm in length. The module is interchangeable with existing fingertip parts, while maintaining the rigidity required for grasping with the robot’s fingers within a defined volume. We also equipped it with a silicone cover with a surface similar to the inner module to provide a compliant contact surface and protect the internal sensing elements during grasping.

A double-sided printed circuit board (PCB) 25 × 30 mm in area was used to integrate a 48-MHz Cortex M0 controller with a Bluetooth low-energy unit (Arduino Nano 33 IoT board) on one side, while the opposite side contained the capacitance to digital converter (CDC) (FDC 2214, Texas Instruments, Dallas, TX, USA) and related circuitry.

To reduce the sensor volume, the electronics for the CDC and the microcontroller (MCU) were integrated on both sides of the PCB. The schematics of the reference boards were reorganized so that a double-sided board could be fabricated within the limited volume. In this process, the inertial measurement unit (IMU) sensor of the Arduino board was removed as it was not essential for the circuitry. This is because the position and angle of the tip can already be calculated from the robot’s fingers by measuring the angles between the robot arm and finger joints.

**Figure 8:**
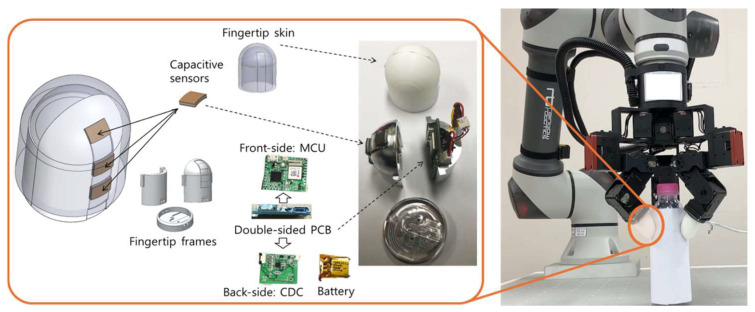
Parts and assembly of the developed module.

Because contact during grasping mainly occurred on the central palmar surface of the fingertip, three capacitive sensors were arranged in a row along the midline of the fingertip. For reliable wire connections, a connecting board was separately made to connect the signal wires of the three sensors. This board and the control board were connected by a flat, flexible cable. A lithium-ion battery was located between the connecting and control boards. As shown in Figure 8, the connecting board, control board, and battery were mounted inside a frame made of AL6061 material, and the three capacitive sensors were attached to the fingertip skin. The module containing the sensors has its own silicone cover. The fingertip skin was fabricated separately using a silicone mold. Uncured silicone (KE-1300T, Shin Etsu, Tokyo, Japan) was poured into the fingertip mold, and an inner pillar was used to create the internal cavity for the fingertip frame and electronics. After curing, the silicone skin was assembled over the sensor-mounted frame. The nominal skin thickness was 2 mm. The silicone skin provided a compliant contact surface and protected the internal sensing elements. However, because it was located between the external object and the capacitive sensors, it also formed part of the mechanical load path. The skin may therefore influence local preload, pressure redistribution, sensitivity, offset, hysteresis, and post-unloading recovery of the embedded sensors. The capacitive sensors were positioned beneath the skin and electrically insulated from the AL6061 frame using insulation tape. As a result, we developed a standalone sensing module for a robotic fingertip that measures the capacitance responses of the three capacitive sensors and transmits the measurements via Bluetooth without an electrical connection to the robotic hand controller. The total weight of the module was 45 g.

To clarify the practical operation of the standalone module, source-side operational timing parameters are presented in Table 2. The firmware was configured to perform capacitance readout and packet generation every 10 ms, corresponding to an update rate of 100 Hz. Within each update period, CDC readout and packet preparation required approximately 6.87 ms, leaving a timing margin of approximately 3.13 ms before the next scheduled update. Because host-side reception depends on the BLE adapter, operating system, logging software, and wireless environment, generalized packet-loss rate and end-to-end BLE latency were not claimed in this study.

## 4. Experimental Results

The grasping experiments were conducted to evaluate whether the assembled fingertip module could provide location-dependent relative capacitance responses during robotic grasping as a system-level functional demonstration of the fully assembled fingertip module. The objective was to confirm that the module could acquire and wirelessly transmit location-dependent tactile responses during actual robotic grasping while operating independently of the robotic hand controller. In this study, independent operation refers to electrical, sensing, and data-transmission independence from the robotic hand controller; it does not refer to autonomous grasp control by the fingertip module. The experiments were not designed to validate grip-force control or to isolate the effects of object mass, compliance, or geometry. In addition, the measured responses were not converted into absolute contact forces, because the assembled fingertip was not recalibrated after installation beneath the silicone skin. Accordingly, the grasping results are interpreted as relative capacitance and local-loading patterns under the tested grasping conditions.

The fingertip module was mounted on one finger of a customized electromotor-driven three-fingered, 11-DoF robotic hand [29] equipped with ROBOTIS XH430-V350 actuators to evaluate its operation during grasping. In the present study, the gripper was modified to mount the proposed tactile fingertip module in place of one original fingertip. The original robotic hand controller generated the finger motion, whereas the developed module was powered and operated independently of the robotic hand controller, and capacitance data were transmitted wirelessly to a separate PC. The purpose of this experiment was to determine whether the module could provide contact-location-dependent relative capacitance responses during grasping, rather than to estimate absolute grasping force. Three cylindrical objects were used: an empty PET bottle, a water-filled PET bottle, and a water-filled aluminum tumbler. These objects were selected as representative objects providing different practical contact and loading conditions. They were not intended to constitute a controlled object-property test set.

**Figure 9:**
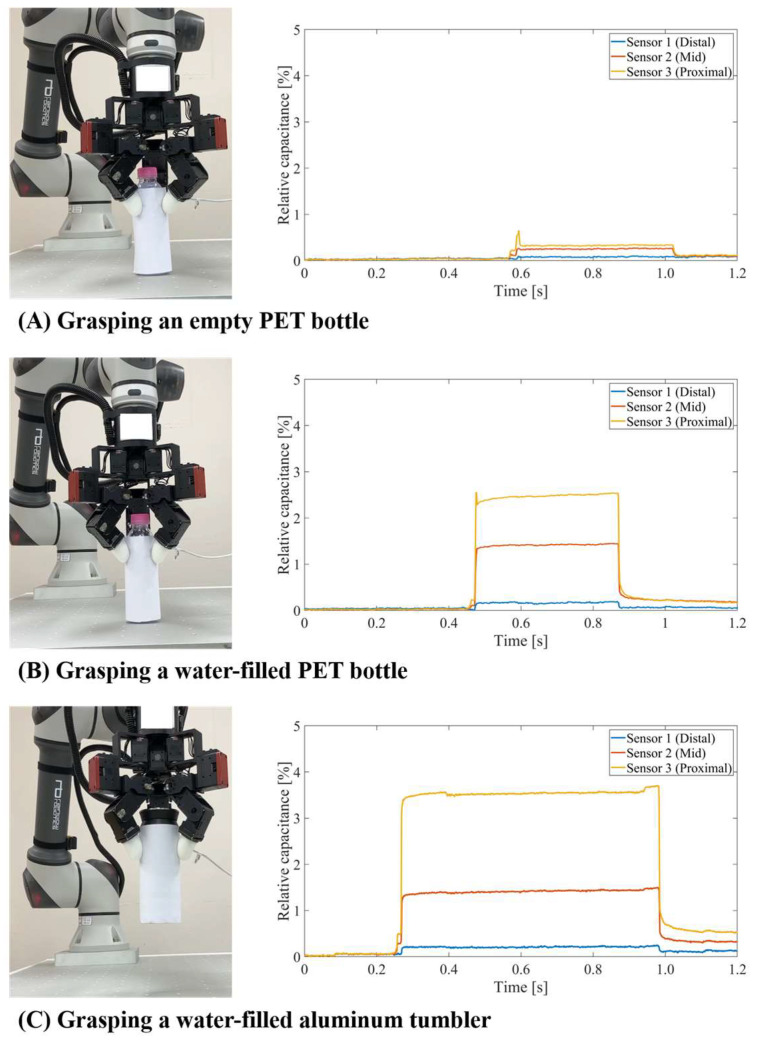
Relative capacitance responses during robotic grasping of three cylindrical objects: (**A**) empty PET bottle, (**B**) water-filled PET bottle, and (**C**) water-filled aluminum tumbler. Sensor 1, Sensor 2, and Sensor 3 correspond to the distal-, mid-, and proximal-position sensors, respectively. The responses are shown as relative capacitance changes, ΔC/C_0_ (%).

The three objects were grasped using conditions sufficient to lift each object without visible slip or crushing. Because the grasp command and contact distribution were not independently controlled, the responses should be interpreted as relative capacitance patterns under representative grasping conditions rather than as quantitative comparisons of object properties. Figure 9 shows the relative capacitance changes of the three sensors during grasping. The distal-position sensor showed only a weak response under the tested grasping configurations, whereas the mid- and proximal-position sensors (i.e., red-colored and yellow-colored ones in Figure 1A, respectively) produced more prominent capacitance changes. This indicates that contact was mainly concentrated in the mid-to-proximal region of the fingertip during the tested grasps. Compared with the empty PET bottle, the water-filled PET bottle produced larger relative capacitance responses, particularly at the mid- and proximal-position sensors. This indicates increased relative local loading in the mid-to-proximal region under the tested grasping condition. Figure 9C shows the change in capacitance when the water-filled aluminum tumbler was grasped. During the aluminum tumbler grasp, the distal- and mid-position sensor responses were comparable to those observed for the water-filled PET bottle, whereas the proximal-position sensor showed a larger capacitance change. This suggests that local loading was more concentrated near the proximal region of the fingertip during the tumbler grasp. This difference may be associated with the grasping condition required to prevent slip, but it should be interpreted as a relative capacitance response rather than a direct measurement of local gripping force.

Table 3 summarizes the details of the relative capacitance responses calculated from the stable grasping intervals in Figure 9. The mid- and proximal-position sensors showed larger responses than the distal-position sensor, indicating that contact loading was mainly concentrated in the mid-to-proximal region of the fingertip during the tested grasps. Compared with the empty PET bottle, the water-filled PET bottle produced larger mid- and proximal-position responses. The aluminum tumbler produced the largest proximal-position response, suggesting increased local loading near the proximal region of the fingertip.

## 5. Discussion and Conclusions

The main contribution of this study is not the introduction of a new capacitive sensing principle, but the integration of capacitive tactile sensing into a compact standalone fingertip module. The proposed module integrates a 3 × 1 flexible capacitive sensor array, CDC-based capacitance readout, microcontroller-based processing, Bluetooth communication, and onboard battery power within a 45 g fingertip-shaped housing. This architecture enables the module to operate electrically independently of the robotic hand controller, while being mounted in place of the original fingertip of the tested hand. The design represents a trade-off between tactile richness and integration complexity. Compared with vision-based tactile fingertips and high-density electronic skins, the proposed module provides lower spatial resolution but reduces the need for cameras, illumination, external acquisition hardware, and multiple sensing channels. Thus, the advantage of the proposed system lies in module-level integration and wireless operation rather than high sensor density or high-resolution tactile imaging.

Such standalone tactile modules may simplify the integration of contact sensing into robotic systems because they reduce the need for external wiring and dedicated data-acquisition hardware. Although stable grasping can be achieved without tactile sensing in some controlled settings, tactile information can provide additional contact cues that may support future grasp monitoring, interaction assessment, and closed-loop control. In the present study, however, we evaluated the module only as a source of relative, location-dependent capacitance responses.

The module we designed and characterized in this study has capacitive sensors that do not directly contact the object grasped by the robot fingertip. The capacitive sensors are covered with a silicone skin to mimic a human fingertip. The skin provides a compliant contact surface and protection, but it also forms part of the mechanical load path and may introduce local preload, pressure redistribution, and recovery effects. Because the skin–sensor coupling was not independently characterized, the post-release baselines in Figure 9 were not analyzed as quantitative recovery or creep metrics. Any post-release baseline offset in the grasping traces may reflect combined contributions from the silicone skin, the skin–sensor interface, object deformation, and sensor/electronics baseline drift. Furthermore, the response of the module is expected to depend on object mass, compliance, geometry, contact area, and grasp configuration. For very light objects, such as feather-like objects, the generated normal contact load may be close to the noise floor of the assembled module, especially if contact occurs away from the three sensing locations. For heavy objects, the capacitance response may increase, but lifting capability is determined by the robotic hand actuation, transmission, friction, and grasp strategy rather than by the sensor module itself. Objects with different geometries may also shift contact toward the side or edge of the fingertip, where the current 3 × 1 centerline sensor arrangement may not provide sufficient spatial coverage. Therefore, daily-object grasping applications would require additional sensing locations, repeated trials, calibrated assembled-fingertip measurements, and controlled evaluation over systematically varied object mass, compliance, shape, and surface properties.

Three capacitive sensors were arranged in a row in the proposed fingertip. This number was determined by considering the number of measurement channels provided by the CDC board and the effective part of the gripper used in the study when it came into contact with an object. However, depending on the dexterity of the robotic finger, additional sensing on the side of the fingertip may be required, or measurements on more channels may be needed for finely detailed area-specific tactile information. The fabricated sensor could also be built with a larger area if needed. In addition to the sensing channels and areas, it is also possible to adjust the weight, battery capacity, and ultimately the overall cost of the module. The CDC used has 28-bit resolution and is relatively expensive. Different CDCs can be used depending on the number of sensors and mid-range resolution required. Similarly, weight savings could be achieved by using lightweight engineering plastic frames instead of AL6061. Although each fingertip module is able to operate independently and multiple modules could be mounted on multiple fingers or on both hands of a bimanual robotic system, multi-finger tactile data fusion, and bimanual grasp-control integration were not evaluated in the present proof-of-concept study.

Creep behavior was not independently characterized in this study. A proper creep test would require sustained constant-load or constant-displacement conditions and separation of the viscoelastic response of the dielectric layer from that of the silicone fingertip skin. Because the assembled module includes a silicone skin between the object and the sensors, creep-like drift during grasping could arise from the sensor dielectric, the skin, the skin-sensor interface, or object deformation. Therefore, we did not attribute the observed post-grasp residual signal to sensor creep. Long-duration constant-load testing of both individual sensors and assembled fingertip modules remains necessary.

The proposed device is a fingertip-shaped sensing module with an internal AL frame, not a thin sensor patch. Thus, grasping loads are expected to be transmitted mainly through the robotic finger structure, mounting interface, and internal frame, while the capacitive sensors and silicone skin provide contact sensing and a compliant contact surface. The robotic hand equipped with the developed module grasped water-filled objects in the present experiments without apparent structural failure, but maximum payload, impact resistance, and long-term mechanical durability were not quantified. For weak contact, the empty PET bottle grasp provides a low-load example in which the object was grasped without crushing while producing small but distinguishable capacitance responses. However, the module detects contact-induced capacitance changes and does not distinguish whether the contact source is a human or an object. If no normal pressure or deformation is applied at the sensing locations, the output is expected to remain near baseline. The robotic grasping experiments were intended as proof-of-concept demonstrations of system-level operation rather than as a practical tactile-perception benchmark. The objective was to verify that the assembled fingertip module could acquire and wirelessly transmit location-dependent relative capacitance responses during actual robotic grasping. The tests were not designed to evaluate subtle contact changes, object classification, slip onset, fine force modulation, or closed-loop grasp adaptation. The three cylindrical objects provided coarse differences in practical contact and loading conditions, but they did not constitute a controlled object set with systematically varied mass, compliance, shape, or surface properties. Therefore, the grasping results demonstrate the feasibility of wireless relative tactile sensing in a robotic gripper, while practical deployment for subtle tactile perception requires calibrated assembled-fingertip measurements, repeated trials, controlled grip commands, and object sets with systematically varied properties.

In this regard, this study has several limitations. First, force–capacitance calibration was performed on the individual capacitive sensors before integration, whereas the robotic grasping experiments were conducted after the sensors had been installed beneath the silicone fingertip skin. Silicone packaging forms part of the mechanical load path and may alter sensor preload, sensitivity, offset, hysteresis, spatial load distribution, and post-unloading recovery. Therefore, the force–capacitance relationship obtained from the individual sensors cannot be directly transferred to the assembled fingertip, and the grasping responses should be interpreted as relative capacitance and local-loading patterns rather than calibrated force measurements. Controlled calibration of the fully assembled fingertip under normal, tangential, and spatially varying contact loads is required before quantitative force estimation can be claimed. Second, lateral-force and shear-interference effects were not evaluated in this study. Because the sensors are embedded beneath a silicone skin, tangential loads may redistribute normal pressure or introduce shear-related artifacts in the capacitance response. Controlled combined normal–tangential loading tests are required to evaluate lateral-force robustness. Finally, closed-loop tactile control, frequency-dependent response, creep behavior, long-term durability, and comprehensive wireless-link validation were not evaluated in this study and remain necessary future work before deployment in high-speed manipulation tasks.

In this study, we developed a standalone capacitive tactile fingertip module for wireless multi-point contact sensing in robotic grasping. The module integrates a 3 × 1 array of flexible capacitive sensors, capacitance conversion circuitry, a microcontroller, Bluetooth communication, and an onboard battery within a compact fingertip-shaped housing. Individual capacitive sensors characterized before module integration showed a near-linear response to normal compression up to 2.5 N and stable short-term response over 100 repeated compression cycles. After integration beneath the silicone skin, the assembled module produced distinguishable relative capacitance changes at the mid- and proximal-position sensors during proof-of-concept robotic grasping tests. Because the assembled fingertip was not independently calibrated under controlled external loading, these responses should be interpreted as relative contact-location and local-loading information rather than quantitative force measurements. Calibration of the assembled fingertip remains necessary for future force-estimation and closed-loop grasp-control applications. The proposed module provides a compact platform for adding wireless tactile sensing to robotic grippers, while assembled-fingertip calibration and closed-loop grasp control remain necessary future work.

## Figures and Tables

**Table 1:** System-level positioning of representative robotic tactile sensing approaches and the proposed module.

Approach	Main Tactile Capability	Integration Limitation Relative to This Work
Fiber-optic tactile fingers and modular attachments [5,6,7,17,18]	Sensitive tactile or sliding information; modular and fiber-compatible tactile structures	Requires optical fibers and interrogation hardware; fingertip-level sensing can be compact, but complete onboard readout, battery, and wireless electronics are generally not integrated inside the fingertip module
Vision-based and optical tactile fingertips [11,12,19,20,21]	Rich spatial, contact, multi-axis, or deformation information	Requires optical components, cameras or optical readout, illumination, image acquisition, and/or calibration pipelines; integration burden is higher than low-channel electrical modules
Distributed tactile arrays and electronic skins [13,22]	Multi-point or high-density tactile information over a contact area	Larger number of sensing elements increases channel count, wiring, readout complexity, and integration burden
Soft high-information fingertip sensors [23]	Large-area soft fingertip sensing and shear-related information	Provides richer deformation/shear information, but requires multi-electrode measurement and modeling; not aimed at compact standalone fingertip electronics
Wireless capacitive gripper sensing [24]	Wireless capacitive proximity/tactile sensing at the gripper level	Demonstrates self-contained wireless capacitive sensing, but targets gripper claws rather than a replaceable robotic fingertip module
This work	3 × 1 flexible capacitive sensor array for relative multi-point contact sensing	Integrates sensors, CDC, MCU, Bluetooth, and onboard battery inside a compact 45 g fingertip module; operates without electrical connection to the robotic hand controller; mounted in place of the tested hand’s original fingertip

**Table 2:** Specifications of the standalone fingertip module.

Parameter	Specification
Number of tactile sensors	3 (3 × 1 array)
Individual sensor size	8 mm × 6 mm
Dielectric material	Ecoflex 00-30
Module diameter	34 mm
Module length	42 mm
Module weight	45 g
PCB size	25 mm × 30 mm
Silicone cover material	KE-1300T
Silicone cover thickness	2 mm
CDC	TI FDC 2214
MCU	SAMD21 Cortex^®^-M0+ 32-bit
Power	Onboard Li-ion battery (50 mAh)
Operating time	71.1 ± 2.3 min (minimum 68 min)
Communication	Bluetooth wireless transmission
Sampling/transmission rate	100 Hz
Capacitance change update cycle	10 ms
CDC readout and packet data preparation time	6.87 ± 0.02 ms
Per-cycle timing margin	3.13 ± 0.02 ms
BLE connection interval	7.5 ms

Note: The sampling and transmission rate indicates the firmware-level sensing and packet-generation rate. It does not guarantee host-side received data rate, packet-loss rate, or end-to-end BLE latency across different receiving systems. The BLE connection interval is a link-configuration parameter, not a measured end-to-end latency.

**Table 3:** Descriptive summary of plateau relative capacitance responses during the proof-of-concept robotic grasping demonstrations.

Object	Relative Capacitance (ΔC/C_0_, %)			Observed Response Pattern
	Distal (Sensor 1)	Mid (Sensor 2)	Proximal (Sensor 3)	
Empty PET bottle	0.064 ± 0.009	0.242 ± 0.006	0.325 ± 0.004	Smallest responses among the tested conditions
Water-filled PET bottle	0.159 ± 0.020	1.445 ± 0.014	2.532 ± 0.035	Higher mid- and proximal-position responses than empty PET bottle
Water-filled Aluminum tumbler	0.207 ± 0.006	1.438 ± 0.009	3.587 ± 0.028	Highest proximal-position response among the tested conditions

Note: Values are reported as the temporal mean ± standard deviation of ΔC/C_0_ (%) over the stable interval of each grasping demonstration. They are provided to summarize the observed response patterns and are not intended for statistical comparison of object properties. No force conversion was applied to the assembled-fingertip responses because the fully assembled module was not calibrated beneath the silicone skin.

## Data Availability

The data presented in this study are available from the corresponding author upon reasonable request.

## Abbreviations

The following abbreviations are used in this manuscript:

BLE	Bluetooth Low Energy
CDC	Capacitance-to-Digital Converter
FBG	Fiber Bragg Grating
IMU	Inertial Measurement Unit
MCU	Microcontroller Unit
PCB	Printed Circuit Board
PET	Polyethylene Terephthalate
